# Pharmacological Spectrum of Substances Derived from *Albizia julibrissin* Durazz

**DOI:** 10.3390/ijms26167778

**Published:** 2025-08-12

**Authors:** Yuji Yang, Chan-Hyuk Kwon, Young-Min Ham, Min Woo Ha

**Affiliations:** 1 Jeju Research Institute of Pharmaceutical Sciences, College of Pharmacy, Jeju National University, 102 Jejudaehak-ro, Jeju-si 63243, Republic of Korea; 2 Seoul Shingil Rehabilitation Medicine Clinic, 162 Shingil-ro, Yeongdeungpo-gu, Seoul 07362, Republic of Korea; 3 Biodiversity Research Institute, Jeju Technopark, Seogwipo, Jeju-si 63068, Republic of Korea; 4 SynBioChem Convergence Institute, Jeju National University, 102 Jejudaehak-ro, Jeju-si 63243, Republic of Korea

**Keywords:** *A. julibrissin*, julibrosides, pharmacological properties, therapeutics

## Abstract

This study aims to systematically investigate the phytochemical and pharmacological characteristics of *Albizia julibrissin* Durazz (*A. julibrissin*), a plant well-regarded in ethnopharmacology. While previous analyses cover *A. julibrissin*, this work provides an updated analysis of recent research, driven by its medicinal potential and the rising interest in its therapeutic uses. Known for its significant medicinal potential, *A. julibrissin* contains a wide range of bioactive compounds, including triterpenoid julibrosides, flavonoids, and lignans. Comprehensive in vitro and in vivo studies across various cell lines and animal models have demonstrated its notable pharmacological attributes, such as antitumor, antidepressant, anxiolytic, anti-obesity, antimicrobial, and antiparasitic effects. To capture recent advancements, a comprehensive search was conducted and scientific literature was indexed, followed by a comparative pharmacological analysis. This review compiles recent research developments from 2004 to 2024, highlighting the potential role of *A. julibrissin* in therapeutic applications for human diseases.

## 1. Introduction

*Albizia* julibrissin Durazz, commonly referred to as the Persian silk tree, is a deciduous plant with a wide distribution in East Asia, Central Asia, Africa, and North America [1]. Traditionally, a decoction of dried *A. julibrissin* stem bark has been used extensively in Asian cultures as a folk remedy to treat insomnia, lethargy, confusion, and diuresis [2]. In local traditional medicine, it has been used for a diverse range of conditions, including depression, wounds, fevers, abscesses, headaches, abdominal pain, diarrhea, coughs, malaria, and parasitic infections [3]. Recent pharmacological investigations have revealed that *A. julibrissin* is an abundant source of bioactive compounds, including triterpenoids, lignans, flavonoids, and saponins, which have been reported to have a wide range of physiological effects, including anti-inflammatory, antioxidant, antitumor, antidiabetic, antifertility, antiemetic, antidepressant, anxiolytic, and immunomodulatory activities [4]. Notably, certain saponins have been demonstrated to have potential utility in vaccine development, exhibiting both cytotoxic activity and immune-adjuvant effects. Furthermore, the extract of *A. julibrissin* has demonstrated efficacy in addressing a range of traumatic conditions, including bruises, ulcers, abscesses, burns, hemorrhoids, and fractures [5], with its antimicrobial and antiparasitic properties being documented in several studies. Recently, preclinical studies have assessed the anti-obesity, antidepressant, antiangiogenic, and immunomodulatory effects of *A. julibrissin*, suggesting that this plant could represent an important source for future functional natural product-based drug development. Given the potential therapeutic importance of *A. julibrissin*, this work aims to provide a comprehensive summary of the phytochemical constituents, pharmacological properties, and cytotoxic effects of this plant, and to suggest promising directions for further research and potential applications. In the following section, we delineate the botanical taxonomy of *A. julibrissin* assessed here (Table 1). Despite its numerous reported benefits, a comprehensive systematic review that synthesizes recent advancements in the pharmacological and phytochemical research on *A. julibrissin* is lacking. As a method of study, SciFinder (American Chemical Society) and Reaxys (Elsevier) were utilized to search all literature, monographs, and patents on *A. julibrissin* and its pharmacological effects. The findings were organized into custom tables presented in the main text.

## 2. Phytochemistry

The phytochemical analysis of *Albizia julibrissin* reveals a diverse array of bioactive compounds with significant pharmacological potential. Triterpenoid saponins, flavonoids, and lignans are particularly noteworthy, each contributing distinct therapeutic effects. Julibrosides, a class of triterpenoid saponins, and various flavonoids and lignans isolated from the plant have demonstrated substantial bioactivity in studies. In the following sections, we will provide detailed descriptions of these major groups of compounds and their specific roles.

### 2.1. Triterpenoid Saponins

Triterpenoids comprise a prominent class of bioactive constituents in *A. julibrissin*, and have been established to have anti-inflammatory, anticancer, immunomodulatory, antidepressant, and neuroprotective effects, as well as cardioprotective, antimicrobial, and antioxidant activities. Triterpenoid saponins are composed of a triterpene aglycone (sapogenin) featuring a backbone of 30 carbon atoms arranged in a four or five-ring structure, linked to one or more sugar moieties via glycosidic bonds. In the case of Julibrosides, which are distinctive triterpenoid saponins derived from Albizia julibrissin, the aglycone component is oleanane, and the attached sugars may vary; these commonly include glucose, rhamnose, galactose, arabinose, fucose, and xylose. The structural diversity resulting from variations in the triterpene skeleton, types and numbers of sugars, and glycosidic linkage patterns contributes to their extensive range of pharmacological applications. Julibroside J8, a major triterpenoid isolated from *A. julibrissin*, has been reported to show varying degrees of antiproliferative activity in six cancer cell lines (BGC-823, Bel-7402, HeLa, PC-3MIE8, MDAMB-435, and LH-60) in vitro [6], and, notably, the findings of some studies have provided evidence to indicate that julibroside derivatives may exhibit anticancer activity by inducing apoptosis or regulating the cell cycle. These compounds are considered to have the potential to modulate immune cells and inhibit the secretion of inflammatory cytokines, thereby exerting immunomodulatory activity. The structural information of the selected terpenoids discussed in this paper, along with julibroside J8, is presented in Table 2.

### 2.2. Flavonoids

**Figure 1:**
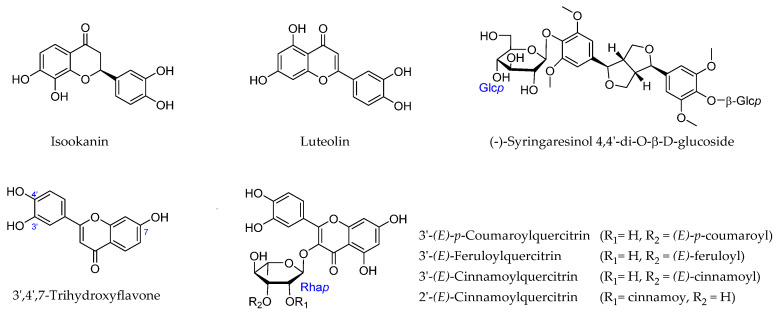
Selected flavonoids derived from *Albizia julibrissin* Durazz.

Flavonoids are among the main bioactive constituents of *A. julibrissin* and are characterized by diverse biological activities, having anti-inflammatory, antifertility, antiproliferative, and sedative properties. Among these compounds, isookanin, luteolin, and [-]-syringaresinol-4-O-d-glucopyranoside, isolated from *A. julibrissin*, have demonstrated radical scavenging-associated antioxidant properties [7]. Moreover, the recently isolated flavonoid glycosides 3′*-(E)-p*-coumaroylquercitrin, 3′-*(E)*-feruloylquercitrin, 3′-*(E)*-cinnamoylquercitrin, and 2′-*(E)*-cinnamoylquercitrin have been found to inhibit the accumulation of fat in 3T3-L1 preadipocytes, thereby indicating potential anti-obesity activity [8]. In addition, 3′,4′,7-trihydroxyflavone has been shown to inhibit the neuronal cell death associated with hydrogen peroxide (H_2_O_2_)-induced oxidative stress, thereby promoting the survival of neurons via its antioxidant activity (Figure 1) [9].

### 2.3. Lignans

**Figure 2:**
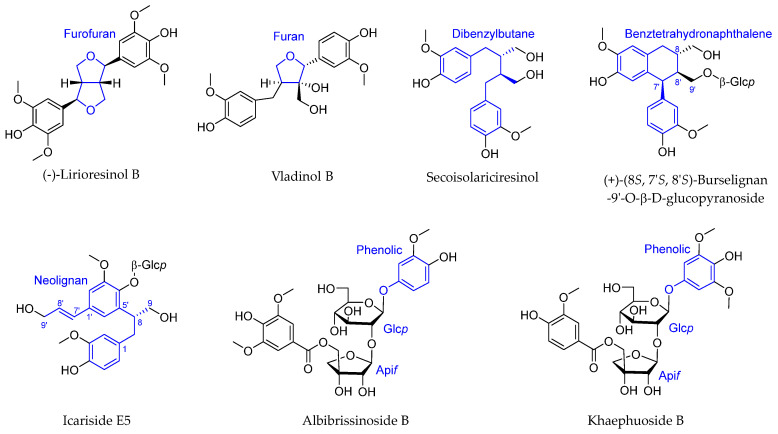
Selected lignans derived from *Albizia julibrissin* Durazz.

Lignanoids are compounds produced via the polymerization of two phenylpropanoid derivatives, members of which have been identified in *A. julibrissin*. These compounds are characterized by a diverse range of bioactivities, including antioxidant, cardiovascular, anticancer, bone disease, anti-inflammatory, and antimicrobial properties. Using a coupled chromatography separation technique, a total of 26 compounds were effectively isolated from a methanolic extract of *A. julibrissin* stem bark with over 90% purity. These included eight furofuran-type, five furan-type, three dibenzylbutane-type, and two bibenztetrahydronaphthalene-type lignans, along with two neolignans and six phenolic derivatives. The anti-inflammatory effects of the isolated analogues were evaluated based on their capacity to inhibit nitric oxide (NO) production in RAW264.7 macrophages of murine origin that had been stimulated with lipopolysaccharide (LPS), with 10 of these compounds exhibiting significant, dose-dependent inhibitory effects (Figure 2) [10].

## 3. Pharmacological Properties

The latest research has been categorized according to the pharmacological functionalities of *Albizia julibrissin*, which are recognized for their diverse and significant therapeutic effects. These findings have been systematically compiled into tables to enhance clarity and ease of reference. Readers are advised to consult these tables to accurately trace and comprehend the extensive range of bioactive effects identified in recent studies. This methodical presentation underscores the multifaceted potential of the plant and offers a comprehensive overview of its promising applications in contemporary medical research.

### 3.1. Antitumor Effect

A substantial body of research has demonstrated the antitumor activity of different saponin compounds isolated from *A. julibrissin*, including julibrosides J8, J28, J21, J29, J30, and J31 (Table 3), the structural configurations of which are presented in Table 2.

#### 3.1.1. Apoptosis-Inducing Effects

Julibroside J8, a triterpenoid saponin isolated from *A. julibrissin*, has been shown to induce apoptosis via the caspase pathway in HeLa cells, which involves the activation and cleavage of the substrate ICAD by caspase-3. The balance between the expression of Bcl-2, Bcl-xL, and Bax played an important role in this process [11]. The tetrasaccharide and trisaccharide portions of a further isolated compound, julibroside J28, have been reported to exhibit significant in vitro antitumor activity against HeLa, Bel-7402, and PC-3M-1E8 cancer cell lines [12], whereas julibroside J21 has shown significant inhibitory activity against the Bel-7402 cancer cell line at a concentration of 10 µg/mL [13]. In addition, when administered at a concentration of 10 µM, julibrosides J29, J30, and J31 have been demonstrated to have significant in vitro antitumor activity against PC-3M-1E8, HeLa, and MDA-MB-435 cancer cell lines, as determined using the SRB and MTT methods [14]. The results of the MTT assay revealed the cytotoxic activity of oleanane-type saponins (julibrosides K-L) and prosapogenins (julibrosides M-O) of A. julibrissin in human lung (A549), colon (HCT116), stomach (BGC-823), and liver (HepG2) cancer cells [15]. Furthermore, a partially purified substance (HaBC18) derived from *A. julibrissin* has been shown to have potent cytotoxic activity against human acute leukemia Jurkat T cells [16].

#### 3.1.2. Antiangiogenic Effects

**Figure 3:**
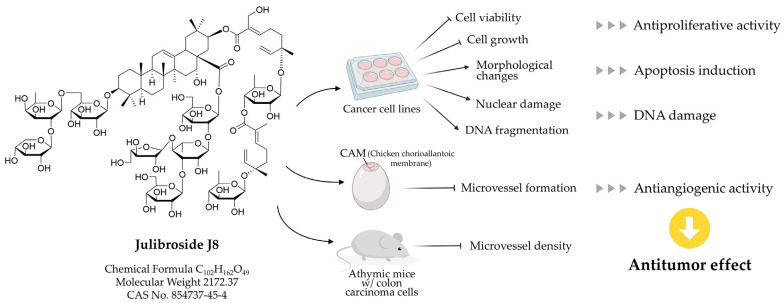
Schematic representation of antitumor effect of Julibroside J8.

Julibroside J8 has been demonstrated to inhibit tumor growth and angiogenesis in vivo, with the latter effect being mediated by hindering the migration of human microvascular endothelial cells (HMEC-1). In a study conducted on solid tumors in athymic mice, it was observed that treatment with this saponin led to a reduction in the microvessel density within tumor tissues, thereby retarding tumor growth. These antiangiogenic properties of julibroside J8 have been demonstrated to be dose-dependent, as evidenced by a series of in vitro models that include prominent features of angiogenesis, such as endothelial cell proliferation, migration, and tube formation. In an in vivo setting, the chorioallantoic membrane (CAM) model has been utilized to demonstrate that julibroside J8 has a significant dose-dependent inhibitory effect on microvessel formation. The antiangiogenic effects of julibroside J8 have also been compared with those of ginsenoside Rg3, a saponin isolated from Panax ginseng, using a xenograft colon cancer model. In the CAM model, julibroside J8 showed the concentration-dependent inhibition of angiogenesis at doses ranging from 10 to 50 μg/egg, with an observed percentage inhibition of approximately 64% at 50 μg/egg, whereas at a concentration of 100 μg/egg, ginsenoside Rg3 was found to exhibit 53% inhibition, thus indicating that julibroside J8 has a more potent antiangiogenic effect than ginsenoside Rg3. Indeed, compared with ginsenoside Rg3, in in vitro and in vivo models, julibroside J8 demonstrated substantial inhibitory effects on angiogenesis at comparatively low concentrations, thereby indicating its potential as a highly effective antiangiogenic and cytotoxic therapeutic agent [17]. In addition, by reducing VEGFR2 activation and its downstream signaling pathways, namely Fak, Akt, and Erk, total saponins derived from *A. julibrissin* (TSAJ) have been demonstrated to inhibit VEGF-induced endothelial cell proliferation, migration, and tube formation, both in vitro and in vivo. These results suggest the potential utility of TSAJ as an antiangiogenic agent, which may inhibit tumor-induced angiogenesis by targeting the VEGF/VEGFR2 signaling pathway (Figure 3) [18].

### 3.2. Antidepressant and Antianxiety Effect

As evidenced by the findings of several studies, antidepressant and anxiolytic effects are among the main pharmacological benefits of *A. julibrissin*, and as shown in Table 4, there have been several studies to date reporting that extracts and key compounds derived from *A. julibrissin* are effective in improving the symptoms of depression and anxiety. Specifically, certain components isolated from *A. julibrissin* have been demonstrated to have an influence on serotonin and GABAa receptors within the brain, thereby producing effects analogous to those observed with the administration of antidepressant and anti-anxiety medications.

*A. julibrissin* has been demonstrated to ameliorate memory impairment induced by short-term insomnia or sleep deprivation. A water-soluble extract of *A. julibrissin* has also been demonstrated to augment the expression of 5-HT1A receptors on serotonergic neurons within the prefrontal cortex and hippocampus of rats [19]. Furthermore, a methylene chloride fraction of *A. julibrissin* (MCAJ) induced antidepressant-like responses. Given that the administration of MCAJ induced a state of mild hypothermia in mice, it is speculated that this MCAJ fraction has 5-HT1A agonist characteristics. Moreover, when assessed using the tail suspension test, the acute administration of MCAJ yielded antidepressant-like effects, whereas the co-administration of a 5-HT1A receptor antagonist (WAY-100635) diminished these antidepressant effects, thereby validating the mediation of this activity via the 5-HT1A receptor system [20]. In addition, (-)-syringaresnol-4-O-β-d-apiofuranosyl-(1→2)-β-d-glucopyranoside, a lignan glycoside isolated from *A. julibrissin*, has been shown to induce anxiolytic-like effects in rats subjected to acute restraint stress in both elevated plus maze and open field tests, and it has been suggested that these effects are associated with the regulation of the hypothalamic–pituitary–adrenal (HPA) axis and the monoamine nervous system [21]. Furthermore, this compound alleviates memory impairment induced by insomnia in fruit flies, indicating its potential efficacy in restoring cognitive function [22]. The findings of another study have revealed that the saponin julibroside C1 isolated from *A. julibrissin* has anxiolytic effects, mediated via the activation of GABAa receptors and the modulation of the HPA axis, thus indicating the potential effects of this compound on the central nervous system [23].

### 3.3. Anti-Obesity Effect

The observed modulatory effects of *A. julibrissin* leaf extract (AJLE) on white adipocyte (3T3-L1) differentiation and the browning process have provided evidence to indicate that this extract has anti-obesity properties, as indicated by its capacity to impede the process of adipogenesis via the modulation of the PPARγ-C/EBPs pathway and the promotion of the differentiation of brown adipocytes by enhancing the expression of UCP-1, PRDM16, and BMP7. The AJLE-induced browning of adipocytes occurs through the regulation of AMPK, p38, and SIRT1 signaling pathways. Moreover, by promoting increases in the mtDNA copy number and UCP-1 expression, AJLE has also been demonstrated to enhance mitochondrial biosynthesis. These findings suggest that AJLE could serve as a potential therapeutic candidate for the treatment of obesity and related metabolic diseases [24]. In addition, flavonol acylglycosides extracted from the flowers of *A. julibrissin* exhibited the capacity to impede fat accumulation and to modulate fat metabolism-related gene expression via the activation of the AMPK pathway [8] (Table 5).

### 3.4. Antibacterial Effect

A methanolic extract of *A. julibrissin* leaves has been found to have potent antimicrobial activity, with minimum inhibitory concentration (MIC) values ranging from 40 to 48 μg/mL against multiple pathogens, including *Bacillus cereus*, *Escherichia coli*, *Enterococcus faecalis*, and *Proteus vulgaris* [25]. Furthermore, a fiber extract of *A. julibrissin* has been demonstrated to have notable antimicrobial activity against a Providencia strain, as revealed by performing disk diffusion assays to evaluate the antimicrobial efficacy of this extract compared with the standard antibiotic streptomycin, which served as a positive control. The findings revealed that at a concentration of 50 μg, *A. julibrissin* fiber samples produced an inhibition zone of 13 mm in diameter, thereby signifying robust antimicrobial activity [26] (Table 6).

### 3.5. Others

In addition to the aforementioned primary biological activities, the constituents of *A. julibrissin* have been shown to possess various pharmacological properties. A multitude of effects have been reported, including the modulation of the immune response, neuroprotective effects, and antiparasitic activity, suggesting the broad therapeutic potential of *A. julibrissin* in addressing various pathophysiological conditions. The studies presented in Table 7 provide evidence for these biological activities and highlight the value of *A. julibrissin* as a natural product resource that may be applied in the regulation of several physiological systems, rather than merely targeting a single disease.

#### 3.5.1. Immune Responses

It has been documented that purified fractions of *A. julibrissin*-derived saponins have demonstrable efficacy as candidate vaccine adjuvants for inactivated H9N2 avian influenza virus vaccines (IH9Vs) in both mouse and chicken models. Both Th1 and Th2 responses have been found to be induced against IH9V, in addition to the enhancement of both antigen-specific humoral and cellular immune responses [27]. Furthermore, in mice, an *A. julibrissin* saponin active fraction (AJSAF) has been demonstrated to augment both antibody and cellular immune responses to porcine reproductive and respiratory syndrome virus (PRRSV), thereby indicating the potential of this fraction to enhance the immunogenicity of PRRSV vaccines. AJSAF has also been demonstrated to induce the production of Th1 (IFN-γ) and Th2 (IL-10) cytokines, with a notable increase in the mRNA expression of the Th1 cytokines IFN-γ and IL-2, as well as their associated transcription factors T-bet and STAT4. Concurrently, the expression of Th2 cytokines IL-4 and IL-10, along with their transcription factors GATA-3 and STAT6, was found to be elevated. These findings accordingly imply the potential applicability of AJSAF as an effective adjuvant that could contribute to enhancing the immune response to vaccination and improving the efficacy of the PRRSV vaccine [28].

#### 3.5.2. Neuroprotective Effects

By directly scavenging free radicals and enhancing the antioxidant status of vomit-stimulated neurons, an extract of *A. julibrissin* flowers demonstrated neuroprotective effects against brain mitochondrial damage, and has also been observed to inhibit vomiting induced by copper sulfate and ipecac in chickens, mediated via peripheral and central mechanisms, thus indicating its anti-emetic properties [29].

#### 3.5.3. Antiparasitic Activity

Leishmaniasis, caused by protozoan parasites., is categorized into three forms, namely, cutaneous, visceral, and mucocutaneous, among which the cutaneous form is the most prevalent. At a concentration of 1000 μg/mL, a methanolic extract of *A. julibrissin* has been found to have a 74.75% inhibitory effect against the promastigote stage of *Leishmania major*, thus providing evidence that indicates the potential utility of *A. julibrissin* extracts in modulating the activity of cytotoxic enzymes implicated in the survival of *Leishmania* [30].

## 4. Conclusions

*Albizia julibrissin* has long been utilized in traditional medicine for the management of symptoms associated with insomnia, anxiety, and depression. Several recent pharmaco-logical studies have provided mechanistic evidence to support its therapeutic use. As described in this review, the constituents of *A. julibrissin* have a diverse range of bioactivities, including antidepressant, anxiolytic, and neuroprotective properties, indicating the potential therapeutic utility of extracts from this plant in the treatment of various pathophysiological conditions, particularly those affecting the central nervous system. The mechanism of action of flavonoids and saponin-like secondary metabolites in *A. julibrissin* can, in many cases, be explained in terms of their interaction with serotonin receptors, GABAa receptors, and other targets, and it is anticipated that the precise mechanisms of action and therapeutic efficacy of these agents will be elucidated, based on the findings of comprehensive molecular-level studies. Recent advances in analytical chemistry, bioinformatics-based mechanistic studies, and drug delivery systems could maximize the pharmacological activity of *A. julibrissin*-derived active constituents and contribute to the development of these into stable and reproducible formulations. In essence, *A. julibrissin* has been rediscovered as a promising candidate for the development of natural product-based drugs with a wide range of applications beyond the scope of traditional medicine, including those for the treatment of neuropsychiatric and inflammatory diseases. In future endeavors, research will focus on profiling the chemical composition of related plant species and uncovering the pharmacological foundations of natural products, including the metabolism of functional secondary metabolites. This work aims to broaden the evidence base and enhance the therapeutic potential of these compounds.

## Figures and Tables

**Table 1:** Taxonomic classification of *Albizia julibrissin* Durazz.

Appearance *	Division	Name
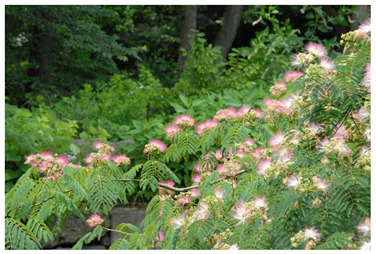	Kingdom	Plantae
	Phylum	Magnoliophyta
	Class	Magnoliopsida
	Subclass	Rosidae
	Order	Fabales
	Family	Fabaceae
	Genus	*Albizia*
	Species	*Albizia julibrissin* Durazz.

* Specifications of resources listed in Biodiversity Research Institute, Jeju Technopark in 2008.

**Table 2:** Selected triterpenoid saponins isolated from *Albizia julibrissin* Durazz.

Core Structure	Name	R^1^	R^2^	R^3^	R^4^
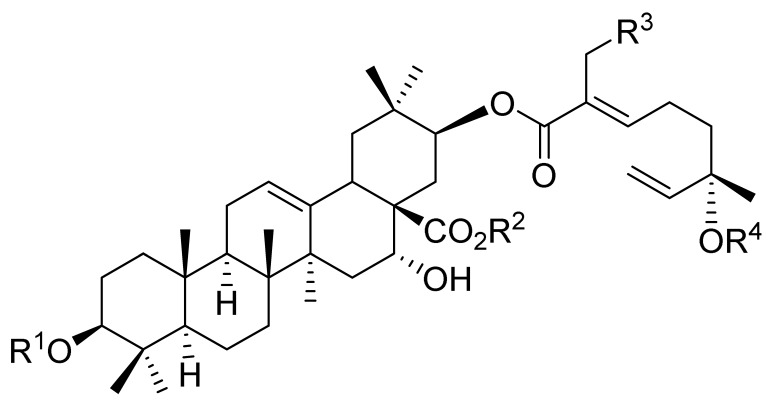	Julibroside J8	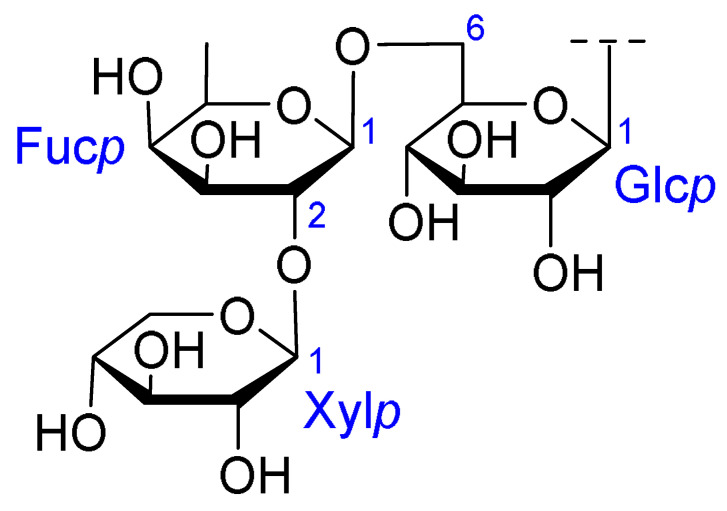	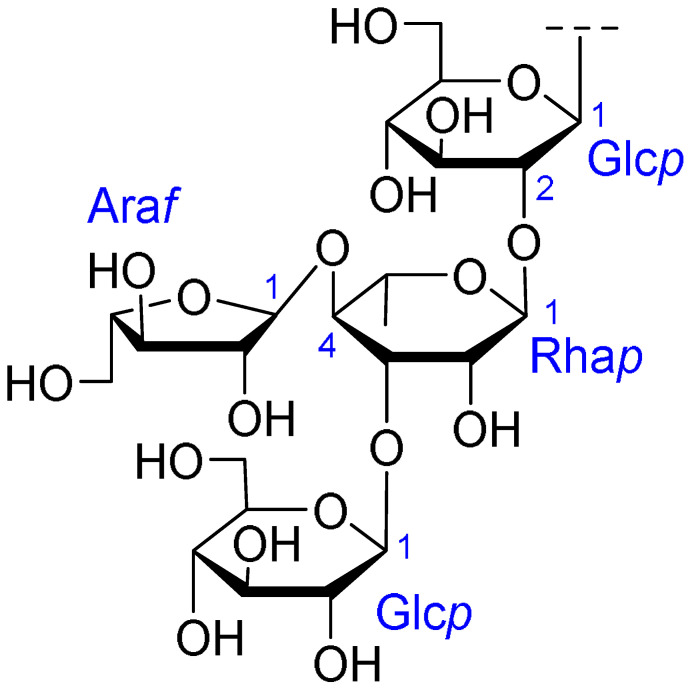	OH	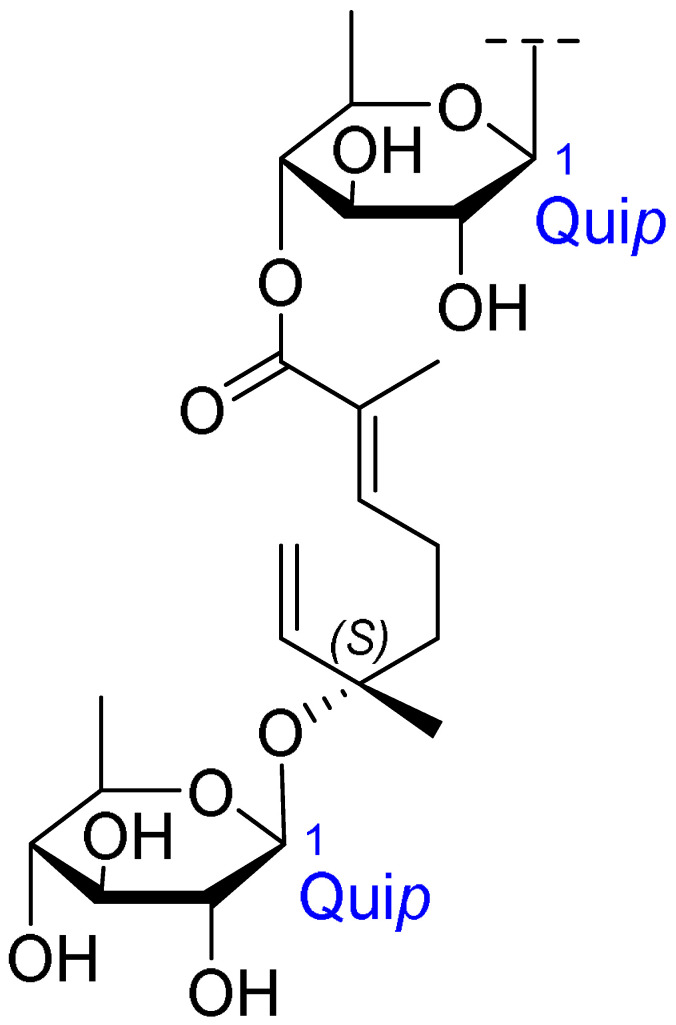
	Julibroside J21	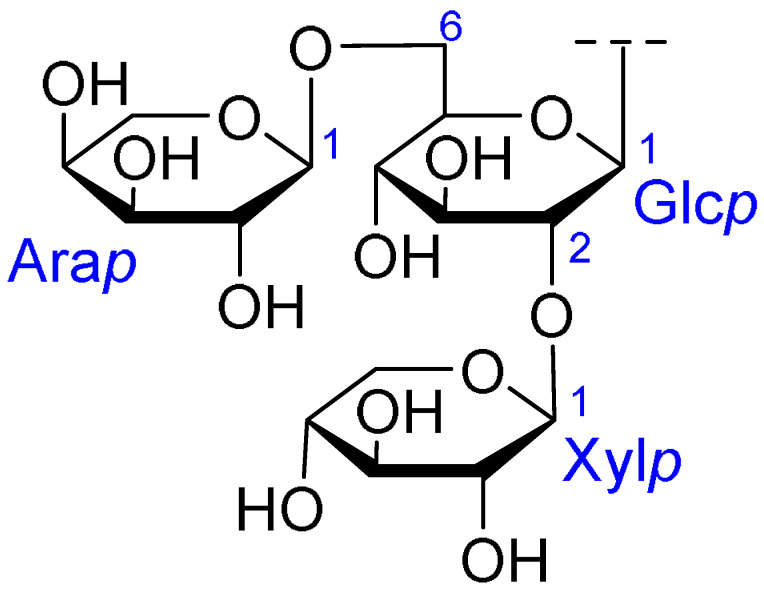		OH	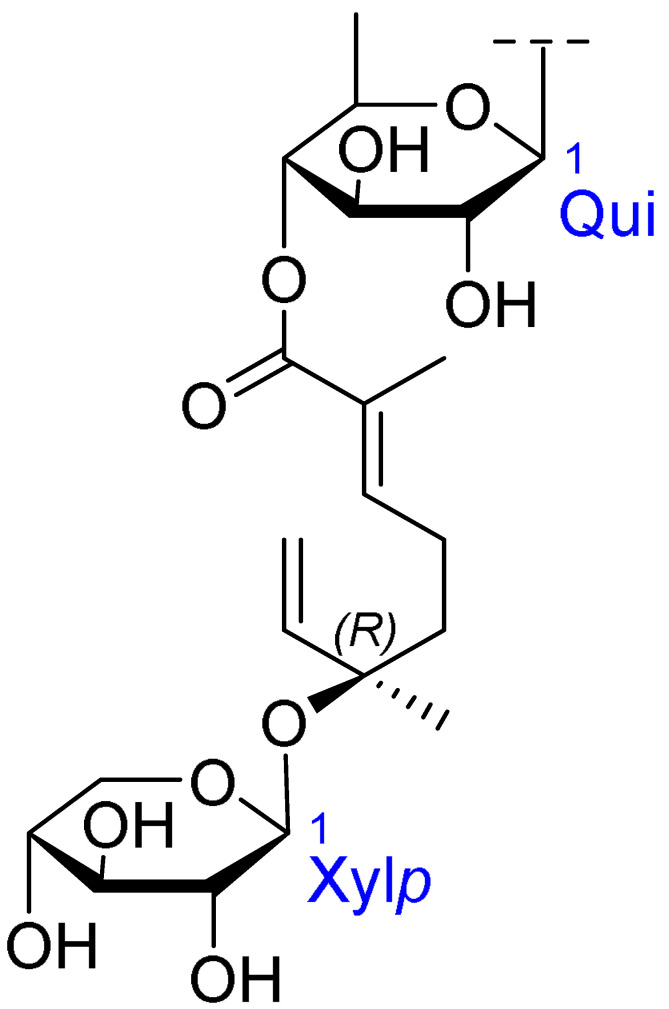
	Julibroside J28	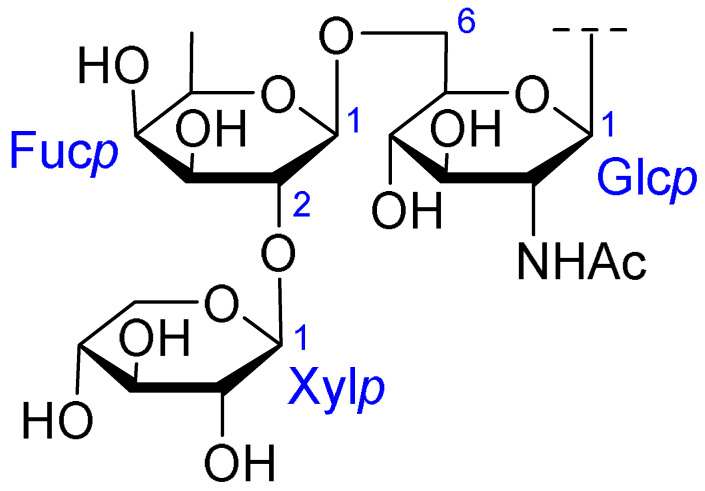		H	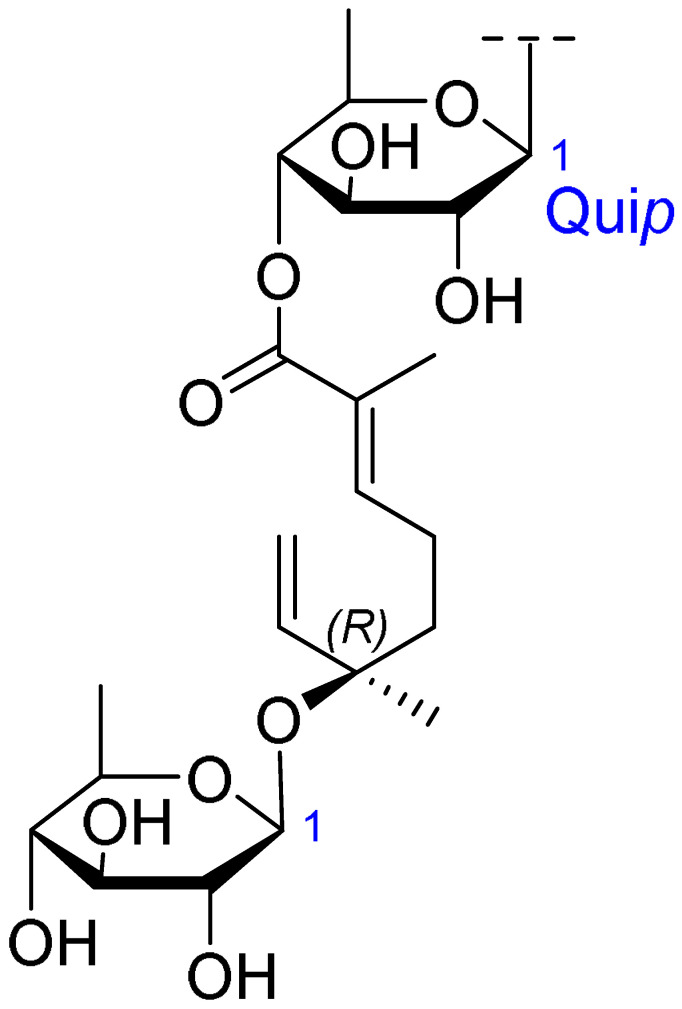
	Julibroside J29			OH	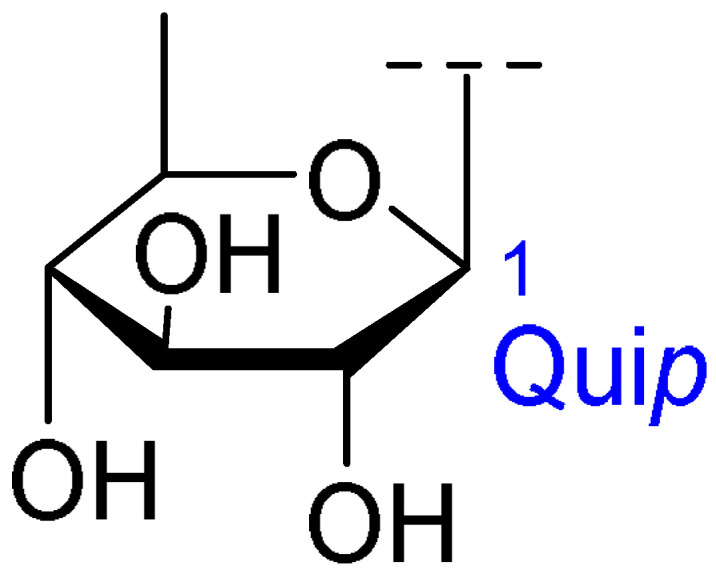
	Julibroside J30			OH	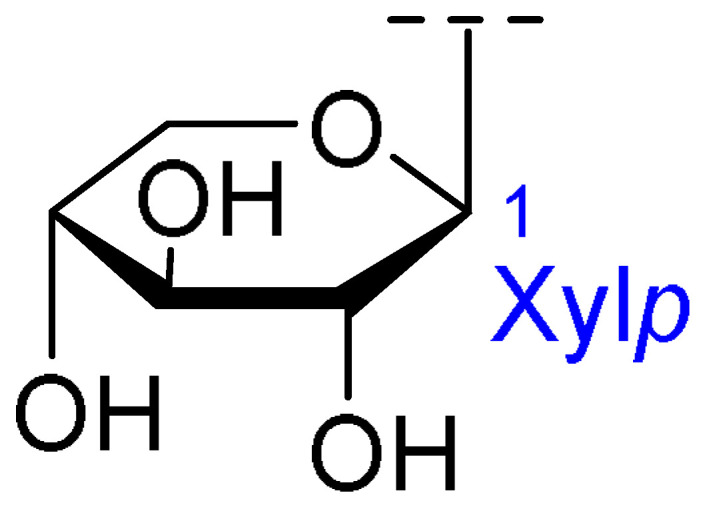
	Julibroside J31	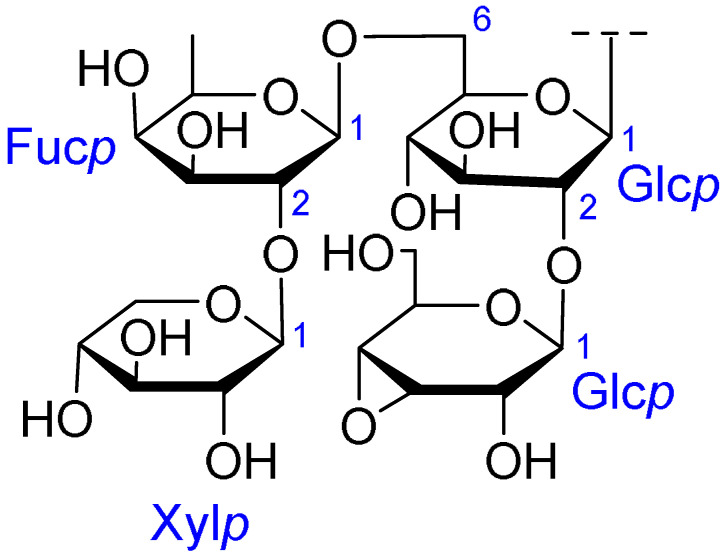		OH	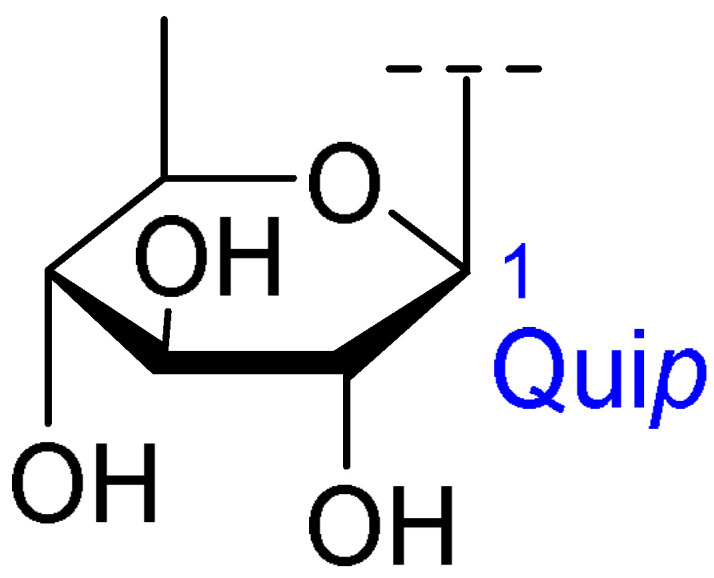

Ara*f*: α-L-arabinofuranosyl; Fuc*p*: α-L-fucopyranosyl; Glc*p*: β-D-glucopyranosyl; Qui*p*: β-D-quinovopyranosyl; Rha*p*: α-L-rhamnopyranosyl; Xyl*p*: β-D-xylopyranosyl

**Table 3:** Antitumor effects of *A. julibrissin.*

Active Ingredient	Model	Mechanism	Ref.
Julibroside J8	HeLa cells	Caspase-3 activation and cleavage of its substrate ICAD, and that the balance between Bcl-2, Bcl-xL and Bax expression.	[11]
Julibroside J28	HeLa, Bel-7402, PC-3M-1E8 cancer cells	Induces apoptosis in tumor cells through caspase-3 activation, ICAD cleavage, and modulation of the Bcl-2/Bax protein balance.	[12]
Julibroside J21	Bel-7402 cell	Induces cytotoxicity in Bel-7402 cancer cells as measured by the SRB assay, potentially through modulation of apoptosis-related pathways.	[13]
Julibroside J29, Julibroside J30, Julibroside J31	PC-3M-1E8, HeLa, MDA-MB-435 cancer cell	Induces cytotoxicity in cancer cells as measured by SRB and MTT assays, potentially through inhibition of cell proliferation and induction of cell death.	[14]
Oleanane-type saponins and prosapogenins from *Albizia julibrissin*	BGC-823, A549, HCT-116, and HepG2 cell	Julibrosides K–L and M–O exhibit cytotoxicity by inducing apoptosis and inhibiting cell proliferation through cell cycle arrest in cancer cells.	[15]
A partially purified substance (HaBC18) from *Albizia julibrissin* Durazz.	Jurkat T cells	Mediated via mitochondria-dependent caspase-3 activation.	[16]
Julibroside J8	HMEC-1/ BALB/C-nu/nu mice were injected subcutaneously with 2 × 10^5^ colon cancer cells (C51)	Inhibits angiogenesis by suppressing the VEGF signaling pathway, specifically impairing endothelial cell proliferation, migration, and tube formation.	[17]
Total saponins of *A. julibrissin*	BALB/c mice, VEGF-induced Ea.hy926 human endothelial cell	Inhibits of VEGFR2 activation and downstream signaling of Fak, Akt, and Erk in vitro and in vivo.	[18]

**Table 4:** Antidepressant and antianxiety effects of *A. julibrissin.*

Active Ingredient	Model	Mechanism	Ref.
Aqueous extract of *Albizzia julibrissin* (AEAJ)	Sprague-Dawley rat	Enhances serotonergic neurotransmission by upregulating 5-HT1A receptor expression in the prefrontal cortex and hippocampus.	[19]
Methylene chloride fraction of *Albizzia julibrissin* (MCAJ)	Male ICR mice	Mediated through the 5-HT1A receptor system.	[20]
(−)-Syringaresnol-4-*O*-β-d-apiofuranosyl-(1→2)-β-d-glucopyranoside (SAG)	Rats stimulated by the elevated plus maze	Enhances inhibitory neurotransmission in the brain through GABA_a_ receptors.	[21]
Aqueous extract of *Albizzia julibrissin*	Sleep-deprived Drosophila	Inhibition of oxidative stress and neuroprotection.	[22]
Julibroside C1	ICR mice stimulated by the elevated plus maze	Enhances of inhibitory neurotransmission through 5-HT1A and GABA_a_ receptors.	[23]

**Table 5:** Anti-obesity effects of *A. julibrissin.*

Active Ingredient	Model	Mechanism	Ref.
*Albizia julibrissin* Leaf extracts (AJLE)	3T3-L1 pre-adipocytes differentiated into white adipocytes	Activates AMPK pathway to inhibit fat differentiation, accumulation, Increases UCP1 and PGC-1a expression to promote browning and energy consumption of white adipocytes.	[24]
Flower of *A. julibrissin*	3T3-L1 cells differentiated into white adipocytes	Flavonol acylglycosides inhibit fat production and accumulation through AMPK pathway activation and regulate fat metabolism gene expression.	[8]

**Table 6:** Antibacterial effects of *A. julibrissin.*

Active Ingredient	Model	Mechanism	Ref.
*A. julibrissin* leaf	Clinically isolated bacterial pathogens (*Bacillus cereus*, *Escherichia coli*, *Entero-coccus faecalis*, and *Proteus vulgaris*)	Inhibits bacterial growth by disrupting cell membrane integrity and metabolic activity, likely mediated by high flavonoid content.	[25]
*A. julibrissin* fibers	Providencia	Inhibits bacterial growth by creating zones of inhibition in disk diffusion assay, possibly through membrane disruption by bioactive phytochemicals.	[26]

**Table 7:** Miscellaneous biological activities of *A. julibrissin.*

Active Ingredient	Model	Mechanism	Ref.
A purified active saponin fraction from the stem bark of *Albizzia julibrissin*	ICR mice, SPF white Leghorn chickens	Enhances humoral and cellular immune responses and induces Th1 and Th2 responses.	[27]
*Albizia julibrissin* saponins (AJSAF)	BALB/c and ICR mice	Enhances both humoral and cellular immune responses by upregulating Th1 (IFN-γ, IL-2, T-bet, STAT4) and Th2 (IL-4, IL-10, GATA-3, STAT6) pathways, thereby boosting PRRSV vaccine immunogenicity.	[28]
Methanolic extract of the flower of *A. julibrissin*	Young chickens induced to vomit with copper sulfate and ipecac	Direct scavenging of free radicals and/or increasing the antioxidant status of the neurons stimulated by emesis.	[29]
Methanolic extract of the of *A. julibrissin*	*Leishmania major* parasites	Likely regulates cytotoxic enzymes involved in parasite survival, exerting anti-leishmanial activity.	[30]
